# Deracemization of Axially Chiral Nicotinamides by Dynamic Salt Formation with Enantiopure Dibenzoyltartaric Acid (DBTA)

**DOI:** 10.3390/molecules181114430

**Published:** 2013-11-21

**Authors:** Fumitoshi Yagishita, Norifumi Kamataki, Kazuma Okamoto, Shota Kanno, Takashi Mino, Hyuma Masu, Masami Sakamoto

**Affiliations:** 1 Department of Applied Chemistry and Biotechnology, Graduate School of Engineering, Chiba University, Yayoi, Inage, Chiba, Chiba 263-8522, Japan; 2 Center for Analytical Instrumentation, Chiba University, Yayoi, Inage, Chiba, Chiba 263-8522, Japan

**Keywords:** dynamic crystallization, chiral salt, deracemization, racemization, axial chrality, nicotinamide, chiral memory, asymmetric synthesis, crystallization, DBTA

## Abstract

Dynamic atroposelective resolution of chiral salts derived from oily racemic nicotinamides and enantiopure dibenzoyltartaric acid (DBTA) was achieved by crystallization. The absolute structures of the axial chiral nicotinamides were determined by X-ray structural analysis. The chirality could be controlled by the selection of enantiopure DBTA as a chiral auxiliary. The axial chirality generated by dynamic salt formation was retained for a long period after dissolving the chiral salt in solution even after removal of the chiral acid. The rate of racemization of nicotinamides could be controlled based on the temperature and solvent properties, and that of the salts was prolonged compared to free nicotinamides, as the molecular structure of the pyridinium ion in the salts was different from that of acid-free nicotinamides.

## 1. Introduction

Axial chirality in aromatic amides is a stereogenic element that arises from the hindered rotation of an aryl-C(=O) single bond [1]. An understanding of how to control the axial chirality of aromatic amides is important, and this has received increasing attention over the past decades. The importance of these chiral elements has been exemplified in attractive asymmetric reactions leading to optically active materials, such as anilides [2–6], *N*-arylimides [7–10], benzamides [11–15] and naphthamides [16–21]. Among a series of aromatic amides, nicotinamide is important because it is an essential element of nicotinamide adenine dinucleotide (NAD) and nicotinamide adenine dinucleotide phosphate (NADP), which are known for their part in biomimetic asymmetric reduction and oxidation in NAD/NADH model systems. The importance of nicotinamides has been demonstrated in both biology and chemistry [22–26]; however, only a few examples of axially chiral nicotinamides have been reported and used for asymmetric synthesis [26–33]. We are interested in the development of convenient methods for preparing axially chiral nicotinamides through dynamic resolution by crystallization.

**Scheme 1:**
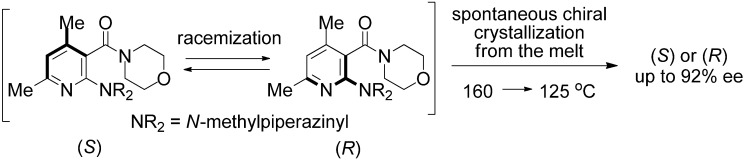
Deracemization of racemic nicotinamide **1** by spontaneous chiral crystallization from the melt.

Spontaneous resolution by crystallization is a powerful tool for obtaining optically active axially chiral materials [34–39]; we recently reported a new example of spontaneous chiral symmetry breaking of an axially chiral nicotinamide, obtaining the product in up to 94% enantiomeric excess (Scheme 1) [40]. The nicotinamide shown in Scheme 1 afforded a conglomerate, and each enantiomer formed enantiomorphic crystals. When the amide was crystallized from the melt in the first racemization, based on C–C(=O) bond rotation, a high *ee* of crystals was obtained with good reproducibility. Total optical resolution of racemic nicotinamide was achieved by a combination of preferential crystallization and racemization.

Optically active materials can be conveniently obtained by this spontaneous chiral resolution; however, the fine methodology involving crystallization is not applicable to all crystalline materials, but is successful only in conglomerate crystals [34]. It is known that conglomerate crystals are formed at a rate of only 10% in organic racemic materials [41,42]. In most cases optically active materials crystallize in chiral fashion. Racemic mixtures may be converted to diastereomers through the formation of chiral salts with enantiopure materials followed by optical resolution by preferential crystallization [43,44]. Dynamic preferential crystallization is particularly useful for amino acids and pharmaceutical reagents [45,46].

We examined the deracemization of nicotinamides by salt formation with enantiopure acids, and succeeded in controlling their axial chirality by deracemization through the growth of crystalline salts. Finally, the chiral acid was removed and a chiral memory of free nicotinamides with axial chirality was generated. To our knowledge, there have been no previous examples of control of axially chiral nicotinamides by salt formation.

## 2. Results and Discussion

**Scheme 2:**
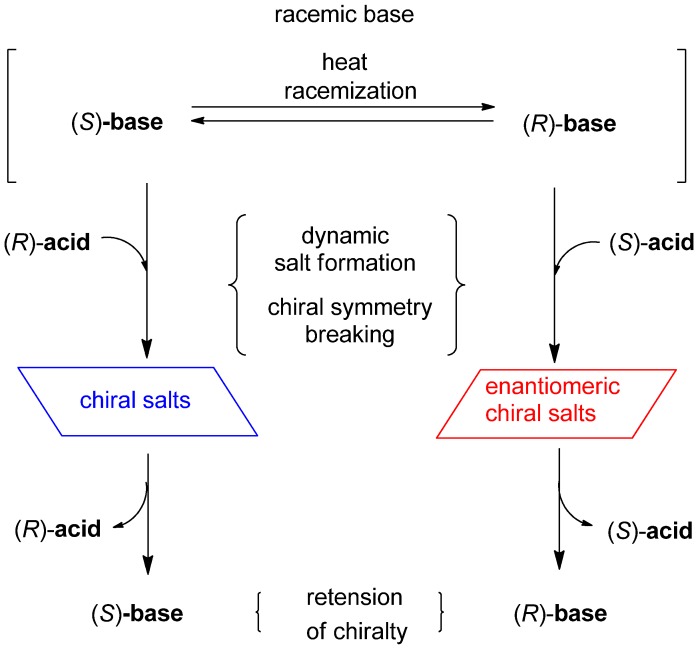
Deracemization by dynamic salt formation.

Deracemization of oily racemic nicotinamides with a basic group was examined by dynamic salt formation with enantiopure acids such as dibenzoyltartaric acid (DBTA), based on the fact that chiral materials tend to crystallize in chiral fashion [34,47,48]. Dynamic racemization through the formation of crystalline salts achieves effective deracemization of the racemic base to give optically active salts. For example, if an enantiopure (*R*)-acid is added to the racemic base under conditions of efficient racemization, and the crystallinity of the (*S*)-base/(*R*)-acid salt is superior to that of the other diastereomeric salt [(*R*)-base/(*R*)-acid], effective dynamic optical resolution can be achieved and the (*S*)-base/(*R*)-acid form will be obtained in enantiopure form with good chemical yield (Scheme 2). Furthermore, the chiral acidic adjuvant can easily be removed by extraction under basic conditions. This is a convenient method of obtaining axially chiral materials. The use of an enantiomeric acid such as an (*S*)-acid gives enantiomeric chiral salts with an (*R*)-base through deracemization in an easy-to-use approach.

**Figure 1:**
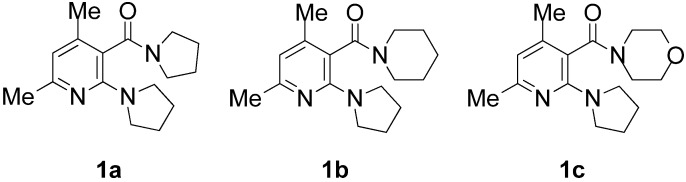
Synthesized oily nicotinamides **1a**–**c**.

**Scheme 3:**
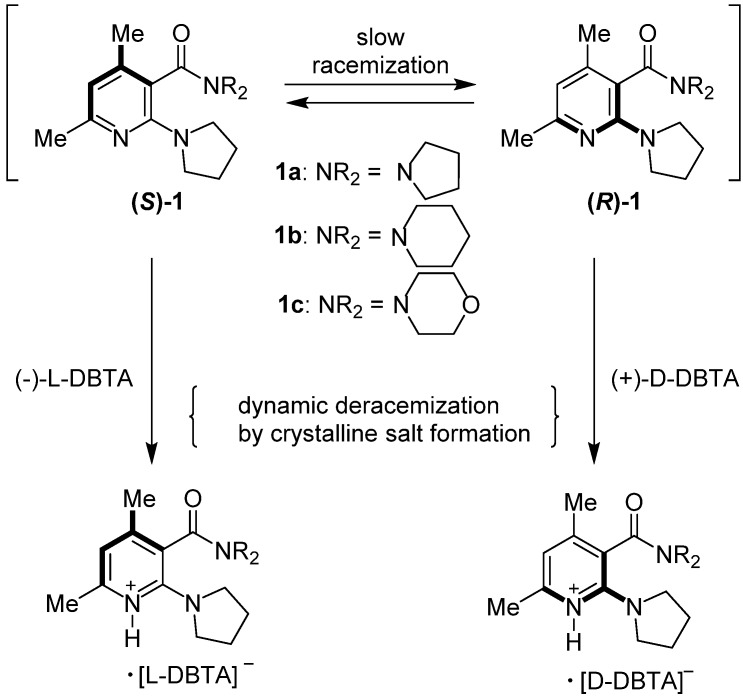
Deracemization of basic nicotinamides **1a**–**c** by crystalline salt formation with enantiopure DBTA.

To examine the deracemization of axially chiral nicotinamides, salt formation was carried out using three oily derivatives, **1a**–**c**, with enantiopure DBTA (Figure 1). When racemic **1a** and an equimolar amount of l-DBTA were dissolved in chloroform and the solvent was removed by an evaporator, a viscous oil was obtained. To analyze the *ee* of **1a** and determine whether deracemization was promoted by salt formation, the salts were dissolved in chloroform and washed with aqueous NaHCO_3_ to remove acidic DBTA. The *ee* value of free **1a** was determined by HPLC using a Daicel CHIRALPAK^®^ AD-H column (Scheme 3). Deracemization was not observed when the materials were mixed without crystallization (Table 1, entry 1). In the cases of **1b** and **1c**, removal of the solvent by evaporation led to a viscous oil, and deracemization was not observed (entries 3 and 6).

**Figure 2:**
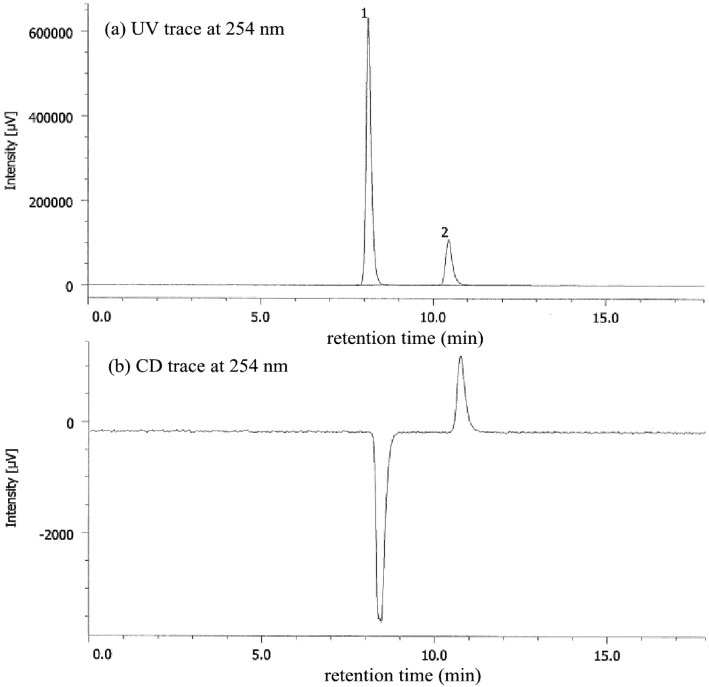
HPLC analysis of **1a** (67% *ee*) after removal of DBTA from the chiral salt by extraction: Daicel CHIRALPAK^®^ AD-H, 0.46 cm ø × 25 cm, UV 254 nm, *t*_R(1)_ = 8.1 min, *t*_R(2)_ = 10.4 min (hexane:EtOH = 90:10, 0.7 mL/min, 20 °C).

In previous experiments involving deracemization by salt formation in the solution phase, it was demonstrated that external chiral stimuli induced a helical sense in a dramatically optically active helical polymer or oligomer [49–54]. In our experiment, the axial chirality of **1a**–**c** could not be controlled in the solution phase because the chiral acid in the salt was located far from the axial chiral auxiliary. In contrast, all of the amides formed crystalline salts with enantiopure DBTA by slow evaporation of the solvent. A chloroform solution of racemic **1a** and an equimolar amount of enantiopure l-DBTA in a test tube was warmed to 60 °C to accelerate the deracemization of **1a**, and removal of the solvent led to the formation of crystalline salts. After all of the solvent had been slowly evaporated off, crystalline salts remained at the bottom of the test tube. The process took about 12 h in all cases. After the removal of DBTA by extraction, the *ee* of **1a** was analyzed by HPLC (Figure 2). The upper part of the figure shows the trace by the UV detector, while the lower trace is that obtained from the CD detector. Five experiments on salt formation of **1a** with l-DBTA gave optically active **1a** in *ee* of 60% to 67% with (−) specific optical rotation (entry 2). This result indicates that deracemization was promoted by dynamic crystalline salt formation. 

**Table 1:** Deracemization of racemic nicotinamides **1a**–**c** by salt formation with (−)-l-DBTA.

Entry	Amide	*ee* (%) *^a^*	Abs. conf *^b^*
1 *^c^*	**1a**	0–5	-
2 *^d^*	**1a**	60–67	(−) *^f^*
3 *^c^*	**1b**	0–6	-
4 *^d^*	**1b**	75–83	(−)-( *S*)
5 *^e^*	**1b**	76–82	(+)-( *R*)
6 *^c^*	**1c**	0–4	-
7 *^d^*	**1c**	51–60	(−)-( *S*)

Notes: *^a^* *Ee* was determined by HPLC using Daicel CHIRALPAK^®^ AD-H column after DBTA was removed by extraction using aqueous NaHCO_3_; *^b^* All nicotinamides **1a**–**c** obtained by the use of l-DBTA showed (−) sign of specific optical rotation. Absolute conformation was established by X-ray analysis; *^c^* Racemic 1 and equimolar amount of l-DBTA was mixed and the solvent was removed by evaporator for each five experiments; *^d^* Crystallization was examined each five times. Each 100 mg of **1a**–**c** and equimolar amount of l-DBTA was used for salt formation. Nicotinamides were recovered quantitatively without loss after salt formation under these conditions; *^e^* Racemic **1b** and equimolar amount of d-DBTA was used for dynamic salt formation; *^f^* Absolute configuration was not determined; however, the **1a** after removing l-DBTA showed the (−) specific rotation.

**Figure 3:**
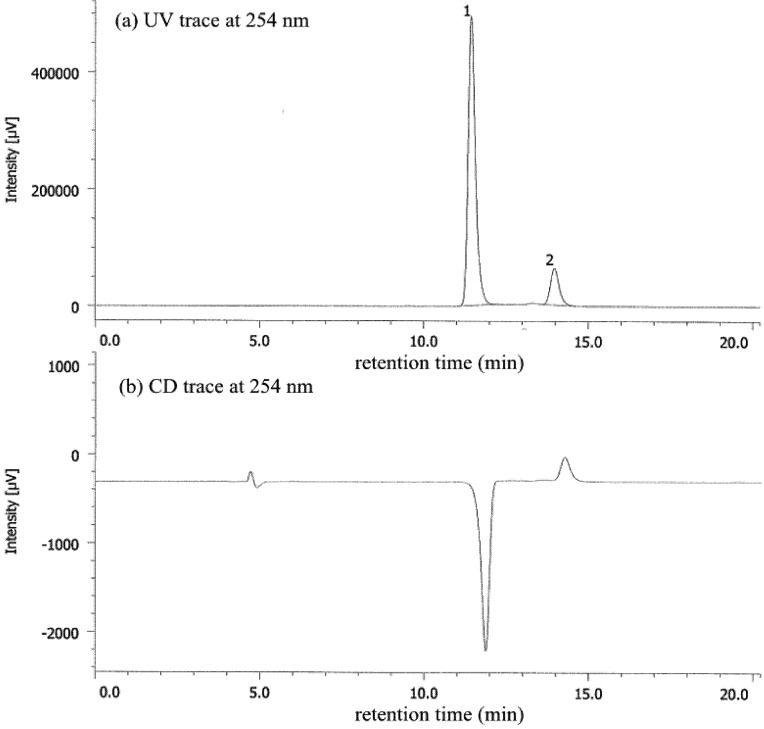
HPLC analysis of **1b** (83% *ee*) after removal of DBTA from the chiral salt by extraction: Daicel CHIRALPAK^®^ AD-H, 0.46 cm ø × 25 cm, UV 254 nm, *t*_R(1)_ = 11.4 min, *t*_R(2)_ = 14.0 min (hexane:EtOH = 95:5, 0.7 mL/min, 20 °C).

In the case of deracemization of **1b** by salt formation with l-DBTA, a better *ee* was obtained (from 75% to 83% *ee*) in five experiments (entry 4). In these experiments, (−)-**1b** was also obtained after removal of l-DBTA, as in the case of **1a**, and the *ee* value was determined by HPLC (Figure 3). When enantiopure (+)-d-DBTA was used for salt formation, (+)-**1b** was obtained with almost the same *ee* value (entry 5). Both enantiomers of **1b** could be easily prepared by selection of the appropriate enantiomeric DBTA. 

**Figure 4:**
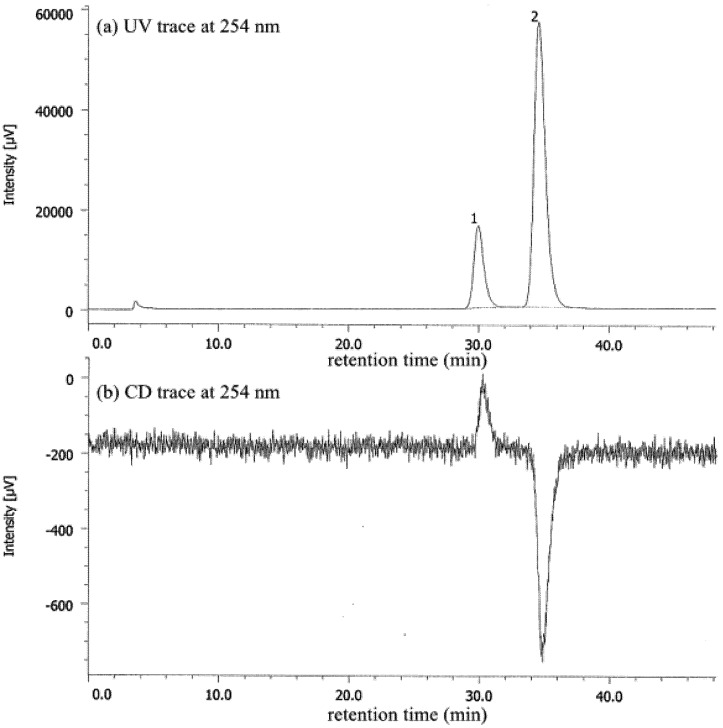
HPLC analysis of **1c** (60% *ee*) after removal of DBTA from the chiral salt by extraction: Daicel CHIRALPAK^®^ AD-H, 0.46 cm ø × 25 cm, UV 254 nm, *t*_R(1)_ = 30.0 min, *t*_R(2)_ = 34.6 min (hexane:EtOH = 95:5, 0.9 mL/min, 0 °C).

Deracemization of **1c** by salt formation with l-DBTA was also successful, and a 51%–60% *ee* of **1c** was obtained (entry 6), which also showed (−) specific optical rotation, after removal of DBTA. Racemization of **1c** was slightly faster than that of **1a** and **1b**, as described below. HPLC analysis was performed at 0 °C (Figure 4). Acid-free nicotinamide **1c** was prepared by extraction of the obtained salt from aqueous NaHCO_3_; therefore, the *ee* value may have decreased during the workup process. Recrystallization from acetone or CHCl_3_ gave enantiopure single crystals for X-ray structural analysis for all salts except that of **1a**/l-DBTA. X-ray analysis of the chiral salts of **1b** and l-DBTA recrystallization from acetone revealed the absolute conformation and the molecular arrangement, which was an orthorhombic crystal system with space group *P*2_1_2_1_2_1_.

**Figure 5:**
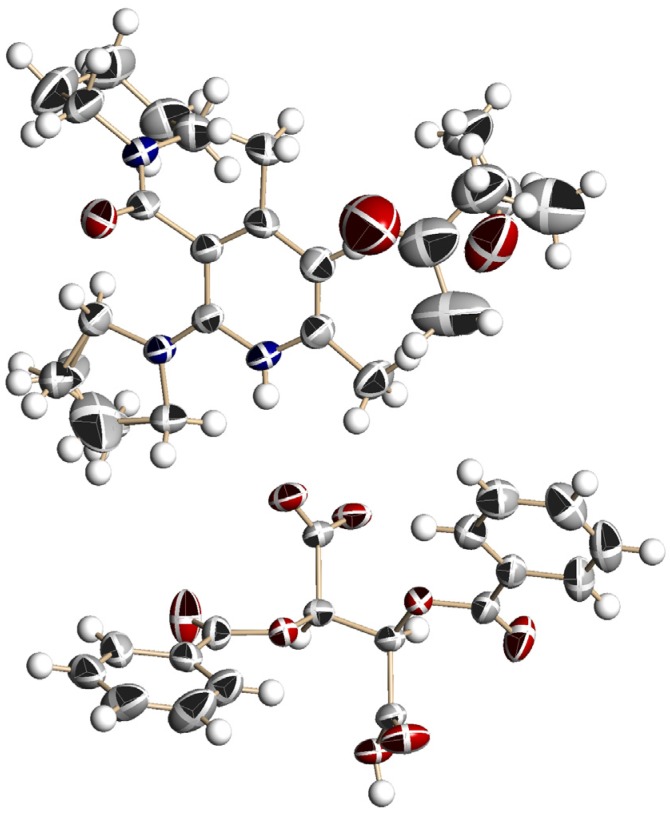
Perspective view of the chiral salt of (a*S*)-**1b** and (−)-l-DBTA with two acetone molecules (acetone molecules are omitted). The ellipsoids are shown as 40% probability. A hydrogen bond between NH (pyridinium) and O(O=)C (l-DBTA) was observed, with a distance of 2.01 Å.

Figure 5 shows the perspective view. The crystal contained two acetone molecules beside **1b** and l-DBTA. Protonation occurred on the nitrogen atom of the pyridine ring, and a near-planar conformation was revealed between the pyridinium ring and the pyrrolidine group at the 2-position. It is known that 2-amino nicotinamides have a distorted conformation between the pyridine ring and the cyclic amino group at the 2-position [40]. These facts indicated that the protonated salts had a different molecular conformation from that of acid-free **1**, and suggest strong conjugation of the lone pair electrons of the nitrogen atom of the pyrrolidine ring with the pyridinium function. In contrast to the planar conformation of the pyrrolidine ring at the 2-position, the amide plane was almost perpendicular to the pyridine ring (dihedral angle: 80.1°). The absolute conformation of the axial chirality of (−)-**1b** was an (*S*)-conformation. The consistency of the absolute conformation of **1b** in the single crystal and the major diastereomeric salt was certified by the HPLC analysis.

**Figure 6:**
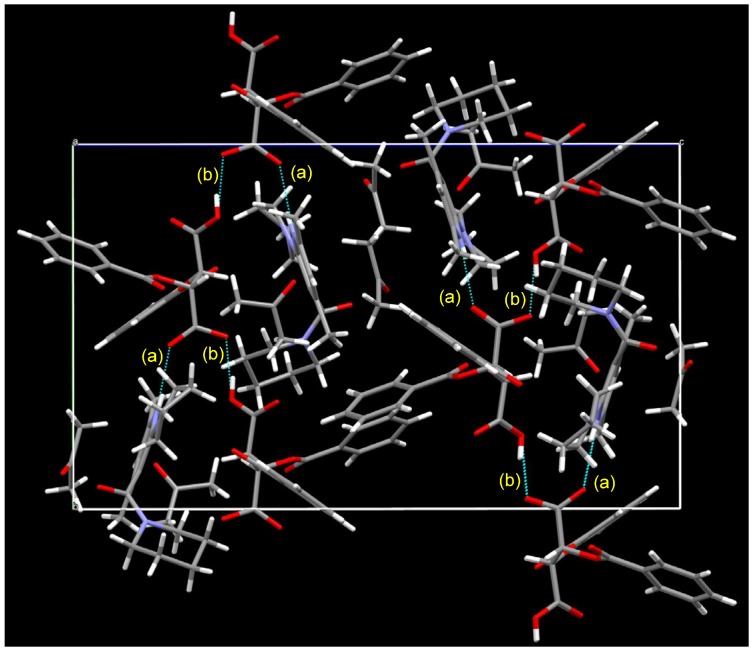
Packing diagram of (a*S*)-**1b**/(−)-l-DBTA/2 acetones from the *a* axis. Two types of hydrogen bond were observed: (a) NH (pyridinium)---O=C (DBTA): 2.01 Å, (b) C(=O)OH (DBTA)---O(O=)C (DBTA): 1.64 Å.

Figure 6 shows the packing diagram for the crystalline salt of **1b** and l-DBTA; a 2_1_ helix is shown along with all axes. Two types of intermolecular hydrogen bond were observed: (a) between NH (pyridinium ion) and O(O=)C (DBTA), with a distance of 2.01 Å, and (b) between two DBTA units, CO_2_H---O(O=)C, with a distance of 1.64 Å.

**Figure 7:**
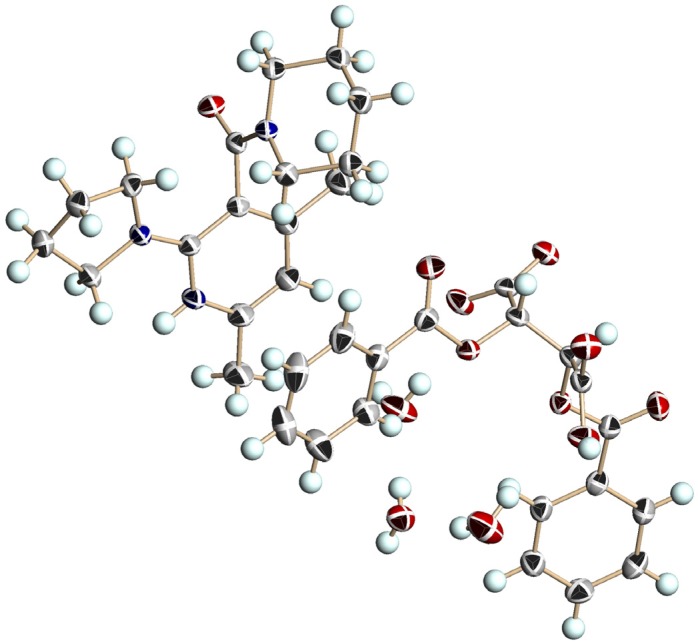
Perspective view of the chiral salt composed of (a*R*)-**1b**, (+)-d-DBTA and 3H_2_O. The ellipsoids are presented as 40% probability. A hydrogen bond between NH (pyridinium) and O_2_C (d-DBTA) was observed with a distance of 2.01 Å.

**Figure 8:**
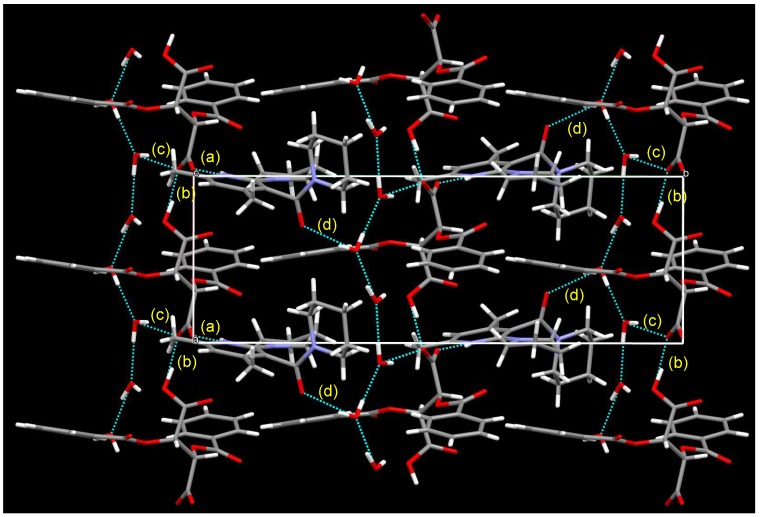
Packing diagram of (a*R*)-**1b**, (+)-d-DBTA and 3H_2_O from the *c* axis. A 2_1_ helix is shown along with the *b*-axis. Four types of hydrogen bond (other than the hydrogen bonds between two H_2_O molecules) were observed: (a) NH(pyridinium)---O=C (DBTA): 2.01 Å, (b) C(=O)OH (DBTA)---O(O=)C (DBTA): 1.53 Å, (c) O(O=)C (DBTA)---HOH: 2.08 Å, (d) NC=O---HOH: 2.08 Å.

Figure 7 shows a perspective view of the enantiomeric salt composed of **1b** and (+)-d-DBTA, with a monoclinic space group *P*2_1_ crystal system, after recrystallization from acetone/water. The crystal contained three H_2_O molecules, and the absolute conformation of **1b** was an (*R*)-configuration. The nicotinamide **1b** obtained by removal of d-DBTA showed (+) specific optical rotation. These facts indicate that the conformation of axial chirality was controlled by salt formation with enantiopure l- or d-DBTA. The chiral crystal (a*R*)-**1b**/(+)-d-DBTA is an enantiomeric form of the crystal of (a*S*)-**1b**/(−)-l-DBTA; however, the two crystal systems are different (*P*2_1_2_1_2_1_ and *P*2_1_) depending on whether the crystallization solvent causes the inclusion of water in the crystal lattice (Figure 8).

**Figure 9:**
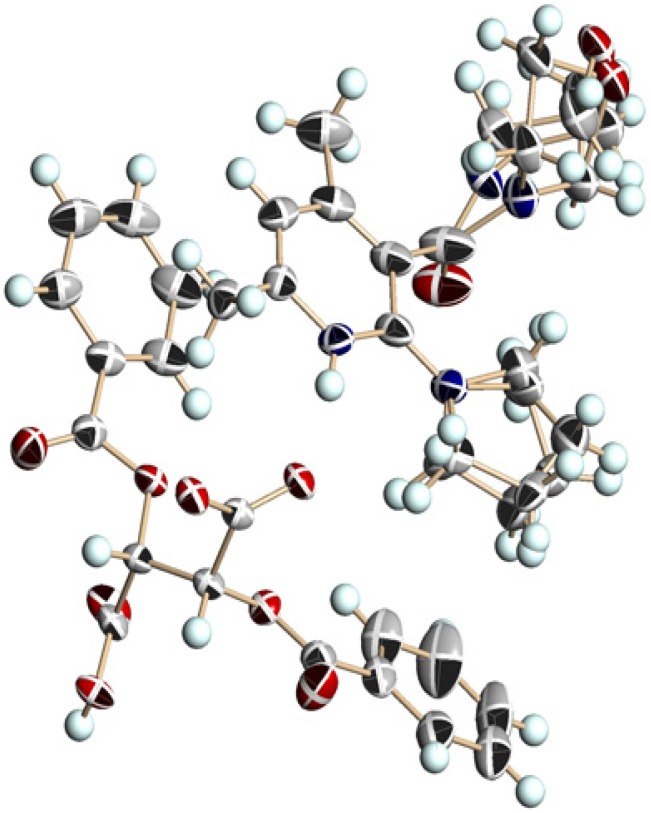
Perspective view of the chiral salt of (a*S*)-**1c** and (−)-l-DBTA. The ellipsoids are presented as 40% probability. A hydrogen bond between NH (pyridinium ion) and O(O=)C (DBTA) was observed with a distance of 1.95 Å.

Recrystallization of chiral salts of **1c** and l-DBTA from CHCl_3_ gave enantiopure single crystals available for X-ray structural analysis. A disordered structure was observed in the morpholine and pyrrolidine groups (Figure 9). Protonation occurred on the nitrogen atom of the pyridine ring, and the pyrrolidine ring on the 2-position adopted an almost planar conformation against the pyridine ring. Furthermore, the absolute conformation of axial chirality was (*S*), which was the same as that of the salt of **1b** and l-DBTA. The consistency of the absolute conformation of **1c** in the single crystal and the major diastereomeric salt was certified by the HPLC analysis and the comparison of the experimental powder diffraction pattern with the calculated powder pattern from single crystal data.

**Figure 10:**
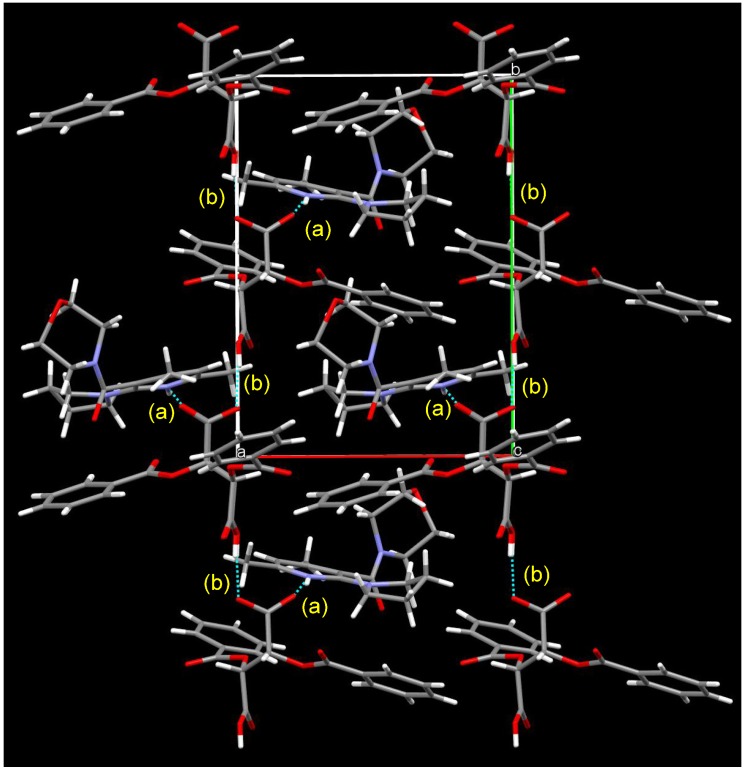
Packing diagram of the chiral salt of (a*S*)-**1c** and (−)-l-DBTA from the *a* axis. A 2_1_ helix is shown along with the *b*-axis. Two types of hydrogen bond were observed: (**a**) NH(pyridinium)---O=C (DBTA): 1.95 Å, (**b**) CO_2_H (DBTA)---O=C (DBTA): 1.68 Å.

Figure 10 shows the packing diagram; a 2_1_ helix is shown along with the *b*-axis. Two kinds of intermolecular hydrogen bond were observed: (a) between NH (pyridinium ion) and O(O=)C (DBTA), with a distance of 1.95 Å, and (b) between two DBTA units (distance 1.68 Å).

For efficient chiral symmetry breaking, fast racemization is required in the crystallization process. In contrast, for practical use of the generated axial chirality in subsequent asymmetric reactions, chiral recognition, *etc.*, chirality should be retained for a long period by slow racemization. Thus, efficient deracemization can have opposing requirements. If the rate of racemization is too low, deracemization cannot be achieved. When it occurs, effective deracemization can be achieved under fast racemization conditions; however, axial chirality may be lost as soon as the salt is dissolved in the solvent. The rate constants (*k*_rac_) and activation parameters for racemization were examined to estimate the conformational stability of **1a**–**c** in three types of solvent—CHCl_3_, MeCN, and methanol. To analyze the racemization of acid-free **1a**, chiral salt **1a**/l-DBTA obtained by deracemization was dissolved in CHCl_3_, and DBTA was removed by extraction with aqueous NaHCO_3_. The rate of racemization was determined based on changes in the specific optical rotation at 20 °C or 30 °C. The same method was used for 1b and 1c. The activation free energies and half-lives are listed in Table 2. 

**Table 2:** Activation parameters for racemization of **1a**–**c** under various conditions *^a^*.

Entry	Nicotinamide	Solvent	*t*_1/2_ *^b^*	*k*_rac_ *^c^*	*ΔG*^‡^ *^d^*
1	1a	CHCl_3_	77.6	1.24 × 10^−6^	25.1
2	1a	MeCN	56.9	1.69 × 10^−6^	24.9
3	1a *^e^*	MeOH	27.7	3.47 × 10^−6^	25.3
4	1b	CHCl_3_	4.7	2.05 × 10^−5^	23.4
5	1b	MeCN	6.2	1.56 × 10^−5^	23.6
6	1b	MeOH	12.3	7.80 × 10^−5^	24.0
7	1b/DBTA *^f^*	MeOH	20.2	4.76 × 10^−6^	24.2
8	1c	CHCl_3_	2.5	3.94 × 10^−5^	23.1
9	1c	MeCN	2.6	3.71 × 10^−5^	23.1
10	1c	MeOH	7.2	1.34 × 10^−5^	23.7
11	1c/DBTA *^f^*	MeOH	9.4	3.06 × 10^−5^	23.2

Notes: *^a^* l-DBTA was removed by extraction from the salts and the rate of racemization of free nicotinamides was measured in several kinds of solvents at 20 °C; *^b^* Half-life in hours; *^c^* Rate of racemization in s^−1^; *^d^* Activation free energy in kcal mol^−1^; *^e^* Measured at 30 °C because of the slow racemization rate at 20 °C; *^f^* Recrystallized chiral salts were dissolved in methanol and the rate of racemization was measured in the presence of l-DBTA.

Among the three nicotinamides **1a**–**c**, 1a had the largest *ΔG^‡^* value (Table 2, entries 1, 4, and 8) because of the most planar pyrrolidine ring. In methanol (a protic and polar solvent), the rate of racemization was lowest in all cases (entries 3, 6, and 10). In the case of **1a**, the rate was measured at 30 °C (entry 3), because racemization was too slow to measure at 20 °C. As shown in entries 7 and 11, salts showed slower racemization than acid-free nicotinamides. This is reasonably explicable on the basis of their conformation. Acid-free 2-alkylaminonicotinamides have a conformation that is twisted between the pyridine ring and the cyclic amino group at the 2-position [40]. However, X-ray analysis (shown in Figure 5, Figure 7 and Figure 9) showed that the pyridinium ion conjugates with the lone pair of electrons of the nitrogen atom at the 2-position. The planar conformation of the cyclic amino group at the 2-position prevents the easy rotation of the amide function at the 3-position.

These findings indicate that the chirality may be controlled by varying the temperature and solvent properties. At high temperatures bond rotation can occur, resulting in fast racemization of these materials, and they may be deracemized to optically active axially chiral nicotinamides by salt formation with enantiopure acid. Furthermore, racemization was suppressed around room temperature, and chirality could be retained for a long period even after removal of the chiral auxiliary DBTA.

Furthermore, a protic solvent such as methanol strongly inhibited racemization; polarity, hydrogen bonding, and solvation by alcohol were important factors that influence the rate of racemization. The effects of solvent polarity may be attributable to the zwitterionic character of the amide group, and solvation of the hydrogen bond with protic solvents reduces the rate of bond rotation.

## 3. Experimental

### 3.1. General

NMR spectra were recorded in CDCl_3_ solution on a Bruker 300 instrument operating at 300 MHz for ^1^H- and ^13^C-NMR spectroscopy. Chemical shifts are reported in parts per million (ppm) relative to TMS as an internal standard. IR spectra were recorded on a JASCO FT/IR-230 spectrometer. Specific rotation was measured using a DIP 370 polarimeter (JASCO). X-ray single crystallographic analysis was conducted using a SMART APEX II or SMART APEX II ULTRA (Bruker AXS).

### 3.2. Preparation of Nicotinamides **1a**–**c**

To a toluene solution containing 2-chloro-4,6-dimethylnicotinic acid (2.00 g, 10.8 mmol) [55] was added thionyl chloride (1.90 g, 16.2 mmol), and the mixture was refluxed for 6 h at 80 °C. After removal of the solvent and excess thionyl chloride *in vacuo*, crude 2-chloro-4,6-dimethylnicotinyl chloride was obtained and used for the subsequent reaction. To a toluene solution of the crude 2-chloro-4,6-dimethylnicotinyl chloride, pyrrolidine (1.83 g, 27.0 mmol) was added dropwise at 0 °C. After the reaction mixture had been stirred for 2 h at room temperature, water and ethyl acetate were added, and the organic layer was extracted in the usual manner. After evaporation of the organic solvent *in vacuo*, the residual mixture was subjected to chromatography on silica gel, and *N*-(2-chloro-4,6-dimethyl-3-pyridinecarbonyl)pyrrolidine was separated in 80% yield. After *N*-(2-chloro-4,6-dimethyl-3-pyridinecarbonyl)pyrrolidine (1.00 g, 4.2 mmol) had been refluxed with pyrrolidine (3.57 g, 42.0 mmol) in an argon atmosphere overnight, water and ethyl acetate were added and the organic layer was extracted in the usual manner. The residual mixture was subjected to chromatography on silica gel and the corresponding nicotinamide **1a** was separated in 70% yield. Other nicotinamides **1b**–**c** were prepared in the same manner. Structures were determined on the basis of spectral data.

*N-(4,6-Dimethyl-2-(1-pyrrolidinyl)-3-pyridinecarbonyl)pyrrolidine* (**1a**). Yellow oil; IR (cm^−1^, neat) 1,622 cm^−^^1^; ^1^H-NMR (CDCl_3_) δ 1.60–1.97 (m, 8H), 2.15 (s, 3H), 2.33 (s, 3H), 2.97–3.03 (m, 1H), 3.20–3.29 (m, 1H), 3.34–3.40 (m, 2H), 3.52–3.68 (m, 4H), 6.30 (s, 1H); ^13^C-NMR (CDCl_3_) δ 19.0, 24.3, 24.7, 25.6, 25.7, 45.1, 47.3, 48.1, 113.2, 114.4, 145.1, 153.4, 156.2, 169.2; EI-MS *m/z* (rel intensity) 273 (M^+^, 74); HRMS (FAB-MS) *m/z* calcd for C_16_H_24_N_3_O 274.1914, found 274.1909.

*N-(4,6-Dimethyl-2-(1-pyrrolidinyl)-3-pyridinecarbonyl)piperidine* (**1b**). Yellow oil; IR (cm^−1^, neat) 1,632; ^1^H-NMR (CDCl_3_) δ 1.39–1.42 (m, 1H), 1.56–1.70 (m, 5H), 1.84–1.94 (m, 4H), 2.15 (s, 3H), 2.34 (s, 3H), 3.02–3.11 (m, 1H), 3.16–3.24 (m, 1H), 3.36–3.57 (m, 5H), 3.96–4.00 (m, 1H), 6.30 (s, 1H); ^13^C-NMR (CDCl_3_) δ 19.1, 24.2, 24.4, 25.5, 25.6, 25.9, 41.9, 47.7, 48.2, 113.1, 113.3, 145.3, 153.8, 156.0, 169.2; EI-MS *m/z* (rel intensity) 287 (M+, 44); HRMS (FAB-MS) *m/z* calcd for C_17_H_26_N_3_O 288.2070, found 288.2067. 

*N-(4,6-Dimethyl-2-(1-pyrrolidinyl)-3-pyridinecarbonyl)morpholine* (**1c**). Yellow oil; IR (cm^−1^, neat) 1,633; ^1^H-NMR (CDCl_3_) δ 1.82–1.93 (m, 4H), 2.17 (s, 3H), 2.34 (s, 3H), 3.11–3.24 (m, 2H), 3.37–3.72 (m, 8H), 3.78–3.85 (m, 1H), 3.96–4.03 (m, 1H), 6.32 (s, 1H); ^13^C-NMR (CDCl_3_) δ 19.1, 24.3, 25.6, 41.4, 46.9, 48.4, 66.5, 66.7, 112.1, 113.5, 145.5, 154.1, 156.6, 169.6; EI-MS *m/z* (rel intensity) 289 (M^+^, 83); HRMS (FAB-MS) *m/z* calcd for C_16_H_24_N_3_O_2_ 290.1863, found 290.1860.

### 3.3. Deracemization of Nicotinamides **1a**–**c** by Crystalline Salt Formation

One hundred mg aliquots of **1** were used for salt formation. A CHCl_3_ solution of racemic nicotinamide **1** and equimolar amount of enantiopure L- or D-DBTA in a test tube was warmed up to 60 °C with stirring until all solvent was slowly evaporated off. Then the crystalline salts remained at the bottom of the test tube, it took about 12 h in all cases.

### 3.4. Determination of the ee Value of **1** after Removing Chiral Acid

After DBTA was removed by extraction, the *ee* of **1** was analyzed by HPLC. Chiral salts were dissolved to a cooled mixture of CHCl_3_ and aq. NaHCO_3_, and the organic layer was separated and washed with cooled water. And the CHCl_3_ solution containing free nicotinamides was analyzed by HPLC using a CHIRALCEL-ADH column.

### 3.5. Crystal Structure of Chiral Salts

Crystal data of chiral salt (a*S*)-**1b**/(−)-L-DBTA/2CH_3_COCH_3_: Colorless prismatic crystals from acetone, C_41_H_51_N_3_O_11_, orthorhombic space group *P*2_1_2_1_2_1_, *a* = 12.43880(10), *b* = 14.1011(2), *c* = 23.5123(3) Å, *V* = 4124.08(9) Å^3^, *Z* = 4, *ρ* = 1.227 g/cm^3^, *μ* (CuKα) = 0.735 mm^−1^, F(000) = 1624. The structure was solved by the direct method of full matrix least squares, where the final *R* and w*R* were 0.0471 and 0.1377 for 7136 reflections, GOF = 1.042, Flack parameter = 0.05(16). CCDC 961138 contains the supplementary crystallographic data for this paper. These data can be obtained free of charge via http://www.ccdc.cam.ac.uk/conts/retrieving.html (or from the CCDC, 12 Union Road, Cambridge CB2 1EZ, UK; Fax: +44 1223 336033; E-mail: deposit@ccdc.cam.ac.uk).

X-ray diffraction analysis data of chiral salt (a*R*)-**1b**/(+)-D-DBTA/3H_2_O: Colorless prismatic crystals from acetone-water, C_35_H_45_N_3_O_12_, monoclinic space group *P*2_1_, *a* = 7.64470(10), *b* = 22.1354(3), *c* = 10.3897(2) Å, *β* = 99.2670(10)°, *V* = 1735.18(5) Å^3^, Z = 2, *ρ* = 1.339 g/cm^3^, *μ* (CuKα) = 0.846 mm^−1^, F(000) = 744. The structure was solved by the direct method of full matrix least squares, where the final *R* and w*R* were 0.0349 and 0.0897 for 5260 reflections, GOF = 1.025, Flack parameter = 0.03(10). CCDC 961139. 

X-ray diffraction analysis data of chiral salt (a*S*)-**1c**/(−)-l-DBTA: Colorless prismatic crystals from hexane-chloroform, C_34_H_37_N_3_O_10_, monoclinic space group *P*2_1_, *a* = 10.6079(8), *b* = 14.4437(11), *c* = 10.6159(8) Å, *β* = 101.1230(10)°, *V* = 1596.0(2) Å^3^, *Z* = 2, *ρ* = 1.348 g/cm^3^, *μ* (MoKα) = 0.100 mm^−1^, F(000) = 684. The structure was solved by the direct method of full matrix least squares, where the final *R* and w*R* were 0.0445 and 0.0883 for 5759 reflections, GOF = 0.953, Flack parameter = −0.30(9). CCDC 961137.

### 3.6. Kinetic Studies for Racemization of **1**

The rate of racemization of **1** was studied in three types of solvent: CHCl_3_, MeCN and methanol. Optically active **1** was prepared from the corresponding chiral salt. The chiral salt was dissolved in CHCl_3_ and the solution was washed with cooled aqueous NaHCO_3_, water and brine. After the organic layer had been dried with anhydrous MgSO_4_, the solvent was evaporated *in vacuo* at room temperature. Changes in the specific rotation of the crude nicotinamides in the three types of solution were monitored at 20 °C or 30 °C. The activation parameters were obtained from the Eyring equation. The first-order kinetic plots of the decay profile of the *ee* values were shown as a plot of ln(*ee*) *versus* time [Equation (1)], and the rate of racemization (*k_rac_*) was calculated from the slope of the line. The free energy barrier (*ΔG^‡^*) for racemization was calculated based on the Eyring equation [Equation (2)]. The half-life was calculated based on Equation (3):

ln(*ee*) = *k*_rac_*t* (1)

*k*_rac_ = (*kT*/*h*)exp(−*ΔG^‡^*/*RT*) (2)

*t*_1/2_ = ln2/2*k*_rac_ (3)

where *k*_rac_: rate of racemization, *h*: Planck constant, *k*: Boltzmann constant, *R*: gas constant, *T*: temperature.

## 4. Conclusions

We have provided a fine example of symmetry breaking to give axial chirality in nicotinamides through the formation of crystalline salts with enantiopure DBTA, which promoted effective dynamic deracemization. The absolute structure of the axial chiral compound became clear, and it was possible to obtain the desired axially asymmetric compound by the selection of enantiopure DBTA as a chiral auxiliary. Furthermore, a kinetic study of racemization showed clearly that the chiral conformation was retained for a considerable time even after removal of the chiral acid. Furthermore, the rate of racemization of nicotinamides could be controlled by selection of the temperature and solvent properties, and that of the salts was prolonged compared to free nicotinamides because the molecular structure of the pyridinium ion in the salts was different from that of acid-free nicotinamides. 

Nicotinamides are well known not only as NAD/NADH model systems, but also as catalysts for many asymmetric reactions. The axially chiral nicotinamides obtained in this work are expected to be useful for subsequent asymmetric reactions and precursors of various types of optically active heterocycle.

## References

[1] Clayden N.A. (1997). New classes of chiral reagents, Auxiliaries, and ligands?. Angew. Chem. Int. Ed. Engl..

[2] Curran P., Qi H., Geib S.J., DeMello N.C. (1994). Atroposelective thermal reactions of axially twisted Amides and imides. J. Am. Chem. Soc..

[3] Guthrie B., Geib S.J., Curran D.P. (2009). Synthesis of highly enantioenriched 3,4-dihydroquinolin-2-ones by 6-exo-trig radical cyclizations of axially chiral R-halo-*ortho*-alkenylanilides. J. Am. Chem. Soc..

[4] Hughes D., Price D.A, Shishkin O., Simpkins N.S. (1996). Diastereoselective enolate chemistry using atropisomeric amides. Tetrahedron Lett..

[5] Kitagawa H.I., Taguchi T., Shiro M. (1997). An Efficient Synthesis of Optically active axially chiral anilide and its application to iodine-mediated asymmetric Diels-Alder reaction. Tetrahedron Lett..

[6] Clayden D., Mitjans L.H.Y. (2002). Lithium-Sulfoxide-Lithium exchange for the asymmetric synthesis of atropisomers under thermodynamic control. J. Am. Chem. Soc..

[7] Curran D.P., Geib S., DeMello N. (1999). Rotational features of carbon-nitrogen bonds in *N*-Aryl maleimides. Atroposelective reactions of o-*tert*-Butylphenylmaleimides. Tetrahedron.

[8] Kondo K., Fujita H., Suzuki T., Murakami Y. (1999). A new chiral axis due to *N*(open-chain imide) -- Ar bond: Unexpected racemization effect of an Acyl group. Tetrahedron Lett..

[9] Shimizu K.D., Freyer H.O., Adams R.D. (2000). Synthesis, resolution and structure of axially chiral atropisomeric *N*-arylimides. Tetrahedron Lett..

[10] Sakamoto M., Iwamoto T., Nono N., Ando M., Arai W., Mino T., Fujita T. (2003). Memory of chirality generated by spontaneous crystallization and asymmetric synthesis using the frozen chirality. J. Org. Chem..

[11] Koide H., Uemura M. (1998). Synthesis of axially chiral *N,N*-diethyl 2,6-disubstituted benzamides utilizing planar chiral (Arene) chromium complexes. Chem. Commun..

[12] Koide H., Hata T., Uemura M. (2002). Asymmetric synthesis of axially chiral benzamides and anilidesbyenantiotopic lithiation of prochiral arene chromium complexes. J. Org. Chem..

[13] Clayden J., Johnson P., Pink J.H., Helliwell M. (2000). Atropisomeric amides as chiral ligands: Using (−)-sparteine-directed enantioselective silylation to control the conformation of a stereogenic axis. J. Org. Chem..

[14] Mai T.T., Branca M., Gori D., Guillot R., Kouklovsky C., Alezra V. (2012). Absolute asymmetric synthesis of tertiary α-amino acids. Angew. Chem., Int. Ed.Engl..

[15] Rios R., Jimeno C., Carroll P.J., Walsh P.J. (2002). Kinetic resolution of atropisomeric amides. J. Am. Chem. Soc..

[16] Thayumanavan S., Beak P., Curran D.P. (1996). Asymmetric deprotonation of *N,N*-dihexyl-l-naphthamides to provide atropisomers of *N,N*-dihexyl-2-alkyl-l-naphthamides. Tetrahedron Lett..

[17] Clayden J., Lai L.W. (1999). Enantioselective synthesis of atropisomeric amides by dynamic resolution: Thermodynamic control with a proline-derived diamine resolving agent. Angew. Chem. Int. Ed. Engl..

[18] Dai W.M., Zhang Y. (2004). Synthesis of atropisomeric 2,8-dioxygenated *N,N*-diisopropyl-1-naphthamides via kinetic resolution under sharpless asymmetric dihydroxylation conditions. Tetrahedron Asymmetry.

[19] Sakamoto M., Unosawa A., Kobaru S., Saito A., Mino T., Fujita T. (2005). Asymmetric photocycloaddition in solution of a chiral crystallized naphthamide. Angew. Chem. Int. Ed. Engl..

[20] Sakamoto M., Kato M., Aida Y., Fujita K., Mino T., Fujita T. (2008). Photosensitized 2 + 2 cycloaddition reaction using homochirality generated by spontaneous crystallization. J. Am. Chem. Soc..

[21] Yagishita F., Mino T., Fujita T., Sakamoto M. (2012). Two-Step asymmetric reaction using the frozen chirality generated by spontaneous crystallization. Org. Lett..

[22] Shaw S.A, Aleman P., Christy J., Kampf J.W., Va P., Vedejs E. (2006). Enantioselective TADMAP-catalyzed carboxyl migration reactions for the synthesis of stereogenic quaternary carbon. J. Am. Chem. Soc..

[23] Kawabata T., Stragies R., Fukaya T., Fuji K. (2003). Remote chirality transfer in nucleophilic catalysis with *N*-(4-pyridinyl)-L-proline derivatives. Chirality.

[24] Spivey A.C., Zhu F., Mitchell M.B., Davey S.G., Jarvest R.L. (2003). Concise synthesis, preparative resolution, absolute configuration determination, and applications of anatropisomeric biaryl catalyst for asymmetric acylation. J. Org. Chem..

[25] Spivey A.C., Leese D.P., Zhu F., Davey S.G., Jarvest R.L. (2004). New atropisomeric biaryl derivatives of 4-aminopyridine-identification of an improved nucleophilic catalyst for asymmetric acylation of *sec*-alcohols. Tetrahedron.

[26] Yamada S., Yamashita K. (2008). Dynamic kinetic resolution of hemiaminals using a novel DMAP catalyst. Tetrahedron Lett..

[27] De Kok P.M.T., Buck H.M. (1985). Regio- and Stereo-selective hydride uptake in model systems related to 3-carbamoyl pyridinium compounds. J. Chem. Soc. Chem. Commun..

[28] De Kok P.M.T., Donkersloot M.C.A., van Lier P.M., Meulendijks G.H.W.M., Bastiaansen L.A.M., van Hooff H.J.G., Kanters J.A., Buck H.M. (1986). Stereoselective hydride uptake in modelsystem related to the redox-couple NAD^+^/NADH. Tetrahedron.

[29] Ohno A., Oda S., Ishikawa Y, Yamazaki N. (2000). NAD(P)^+^-NAD(P)H Models. 90. Stereoselection controlled by electronic effect of a carbonyl group in oxidation of NAD(P)H analog. J. Org. Chem..

[30] Mikata Y., Mizukami K., Hayashi K., Matsumoto S., Yano S., Yamazaki N., Ohno A. (2001). NAD/NADH models with axial/central chiralities: Superiority of the quinoline ring system. J. Org. Chem..

[31] Ishichi Y., Ikeura Y., Natsugari H. (2004). Amide-based atropisomers in Tachykinin NK1-receptor antagonists: Synthesis and antagonistic activity of axially chiral *N*-benzylcarboxamide derivatives of 2,3,4,5-tetrahydro-6Hpyrido[2,3-b][1,5]oxazocin-6-one. Tetrahedron.

[32] Kanomata N., Nakata T. (2000). A compact chemical miniature of a holoenzyme, coenzyme NADH linked dehydrogenase. Design and synthesis of bridged NADH models and their highly enantioselective reduction. J. Am. Chem. Soc..

[33] Kanomata N., Mishima G., Onozato J. (2009). Synchronized stereocontrol of planar chirality by crystallization-induced asymmetric transformation. Tetrahedron Lett..

[34] Jacques J., Collet A., Wilen S.H. (1981). Enantiomers, Racemates, and Resolutions.

[35] Pincock R.E., Perkins R.R., Ma A.S., Wilson K.R. (1971). Probability distribution of enantiomorphous forms in spontaneous generation of optically active substances. Science.

[36] Kondepudi D.K., Laudadio J., Asakura K. (1999). Chiral symmetry breaking in stirred crystallization of 1,1′-binaphthyl melt. J. Am. Chem. Soc..

[37] Sakamoto M., Utsumi N., Ando M., Saeki M., Mino T., Fujita T., Katoh A., Nishio T., Kashima C. (2003). Breaking the Symmetry of Axially Chiral *N*-Aryl-2(1*H*)-pyrimidinones by Spontaneous Crystallization. Angew. Chem. Int. Ed.Engl..

[38] Sakamoto M., Yaghishita F., Ando M., Sasahara Y., Kamataki N., Ohta M., Mino T., Kasashima Y., Fujta T. (2010). Generation and amplification of optical activity of axially chiral *n*-(1-naphthyl)-2(1*h*)-pyrimidinethione by crystallization. Org. Biomol. Chem..

[39] Sakamoto M., Mino T., Mastai Y. (2012). Asymmetric Reaction Using Molecular Chirality Controlled by Spontaneous Crystallization. Advances in Crystallization Processes.

[40] Yagishita F., Okamoto K., Kamataki N., Kanno S., Mino T., Kasashima Y., Sakamoto M. (2013). Chiral symmetry breaking of axially chiral nicotinamide by crystallization from the melt. Chem. Lett..

[41] Sakamoto M. (2007). Spontaneous chiral crystallization of achiral materials and absolute asymmetric photochemical transformation using the chiral crystalline environment. J. Photochem. Photobiol. C.

[42] Koshima H., Matsuura T. (1998). Chiral crystallization of achiral organic compounds: Generation of chirality without chiral environment. J. Syn. Org. Chem..

[43] Kozma D. (2002). CRC Handbook of Optical Resolution via Diastereomeric Salt Formation.

[44] Nohira H., Sakai K., Toda F. (2005). Optical Resolution by Means of Crystallization. Enantiomer Separation.

[45] Coquerel G. (2007). Preferential crystallization. Top. Curr. Chem..

[46] Yoshioka R. (2007). Racemization, optical resolution and crystallization-induced asymmetric transformation of amino acids and pharmaceutical intermediates. Top. Curr. Chem..

[47] Collet A. (1999). Separation and purification of enantiomers by crystallization methods. Enantiomer.

[48] Brands K.M.J., Davies A.J. (2006). Crystallization-Induced diastereomer transformations. Chem. Rev..

[49] Yashima E., Maeda K., Okamoto Y. (1999). Memory of macromolecular helicity assisted by interaction with achiral small molecules. Nature.

[50] Miyagawa T., Furuko A., Maeda K., Katagiri H., Furusho Y., Yashima E. (2005). Dual memory of enantiomeric helices in a polyacetylene induced by a single enantiomer. J. Am. Chem. Soc..

[51] Ousaka N., Inai Y., Kuroda R. (2008). Chain-Terminus triggered chiral memory in an optically inactive 310-helical peptide. J. Am. Chem. Soc..

[52] Pijper D., Jongejan M.G.M., Meetsma A., Feringa B.L. (2008). Light-Controlled supramolecular helicity of a liquid crystalline phase using a helical polymer functionalized with a single chiroptical molecular switch. J. Am. Chem. Soc..

[53] Lautrette G., Kauffmann B., Ferrand Y., Aube C., Chandramouli N., Dubreuil D., Huc I. (2013). Structure Elucidation of Host-Guest Complexes of Tartaric and Malic Acids by Quasi-Racemic Crystallography. Angew. Chem. Int. Ed..

[54] Ferrand Y., Kendhale A.M., Kauffmann B., Grelard A., Marie C., Blot V., Pipelier M., Dubreuil D., Huc I. (2010). Diastereoselective encapsulation of tartaric acid by a helical aromatic oligoamide. J. Am. Chem. Soc..

[55] Dyadyuchenko L.V., Strelkov V.D., Mikhailichenko S.N., Zaplishny V.N. (2004). Synthesis of some halogen-and nitro-substituted nicotinic acids and their fragmentation under electron impact. Chem. Heterocycl. Comp..

